# Milk fat globule membrane coating of large lipid droplets in the diet of young mice prevents body fat accumulation in adulthood

**DOI:** 10.1017/S0007114516001082

**Published:** 2016-04-04

**Authors:** Annemarie Baars, Annemarie Oosting, Eefje Engels, Diane Kegler, Andrea Kodde, Lidewij Schipper, Henkjan J. Verkade, Eline M. van der Beek

**Affiliations:** 1 Danone Nutricia Research, 3584 CT Utrecht, The Netherlands; 2 Department of Pediatrics, Beatrix Children’s Hospital, University Medical Centre Groningen, University of Groningen, 9713 GZ Groningen, The Netherlands

**Keywords:** Metabolic programming, Dietary lipids, Infant nutrition, Milk fat globule membrane, Obesity, Lipid structure

## Abstract

Epidemiological studies have demonstrated protective effects of breast-feeding on childhood obesity. Differences between human milk and infant milk formula (IMF) in dietary lipid structure may contribute to this effect. In our mouse model, feeding a diet containing large lipid droplets coated with phospholipids (PL) (Nuturis^®^; PL of milk fat globule membrane (MFGM) fraction origin) in early life protected against excessive body fat accumulation following a diet challenge in adult life. We now set out to determine the relevance of increased droplet size and/or MFGM lipid droplet coating to the observed anti-obesogenic effects in adult life. From day 16 to 42, male mouse pups were exposed to diets with small (S) or large (L) lipid droplets (0·3 *v*. 2·9 µm average mode diameter, respectively), either without MFGM or with MFGM coating around the lipid droplet, resulting in four groups: S (control diet), L, S^coating^ and L^coating^ (Nuturis^®^ IMF diet). Mice were subsequently challenged with a Western-style diet until dissection at postnatal day 98. A non-challenged group served as reference (REF). We repeatedly determined body composition between postnatal day 42 and 98. At day 98 plasma and gene expression measurements were performed. Only the Nuturis^®^ IMF diet (L^coating^) in early life containing MFGM-coated large lipid droplets reduced body fat mass to a level comparable with the REF group. These data support the notion that the structural aspects of lipids in human milk, for example, both lipid droplet size as well as the MFGM coating, may contribute to its reported protective effect against obesity in later life.

Breast-feeding is associated with a moderate, but consistent, reduction in the risk of childhood obesity and metabolic disease in later life^(^ ^1^ ^–^ ^5^ ^)^. Specific aspects of breast-feeding such as feeding behaviour and its unique nutritional composition may contribute to this long-term protective effect. Human milk (HM) contains several biologically active compounds such as oligosaccharides, hormones and growth factors that are not present in infant milk formula (IMF), supporting optimal growth and development^(^ ^6^ ^–^ ^9^ ^)^. Moreover, HM lipids have a distinct physical structure composed of large lipid droplets (average mode diameter of 4 µm) consisting of a TAG core enveloped by a tri-layer of membranes, the native milk fat globule membrane (MFGM)^(^ ^10^ ^–^ ^12^ ^)^. MFGM components are secreted by the epithelial cells of the mammary gland and consist of phospholipids and sphingolipids, membrane-specific proteins, enzymes, cholesterol, glycoproteins and vitamins. In contrast, the diameter of lipid droplets in standard IMF is about 0·3–1·0 μm and is not surrounded by a membrane, due to the industrial processing procedures to achieve stable and reproducible end products. The IMF fat globules mainly consist of TAG derived from plant oils and have milk proteins adhering to the surface, with or without small amounts phospholipids (PL)^(^ ^13^ ^–^ ^16^ ^)^.

Several studies have indicated that dietary exposure to a MFGM lipid-containing product may have beneficial effects. Clinical intervention studies with MFGM products in adults have shown long-term anti-carcinogenic effects, hypocholesterolaemic effects, suppression of multiple sclerosis and improved learning and cognitive performance after consuming the MFGM extract (reviewed by Spitsberg^(^ ^17^ ^)^). Dietary exposure to MFGM lipid product in early life may have beneficial effects on growth and (cognitive) development in rodents and infants^(^ ^18^ ^,^ ^19^ ^)^. Timby *et al*.^(^ ^19^ ^,^ ^20^ ^)^ showed that infants fed a low-energy, low-protein, MFGM-enriched formula showed cognitive development and serum cholesterol concentrations more similar to those of breast-fed infants at 12 months of age as compared with a standard IMF. The composition of MFGM has been shown to affect pancreatic gastric lipase activity and lipase interaction, cholesterol availability, lipid absorption and absorption of bile salts^(^ ^13^ ^,^ ^14^ ^,^ ^19^ ^)^. However, no growth or associated metabolic effects of MFGM have so far been reported in clinical studies.

We hypothesised that in particular the physical structure of dietary lipids in early life contributes to a healthy development of body composition at longer term via effects on growth and metabolic development during infancy. It has been clearly shown that dietary lipid structure affects lipolysis and the metabolic fate of fatty acids (FA) due to distinct differences in lipid digestion and absorption kinetics^(^ ^21^ ^,^ ^22^ ^)^. Any difference in postprandial lipid handling can impact lipid availability for the development of metabolic organs. This in turn could programme metabolic homoeostasis, energy balance and metabolic response with potential impact on later life health.

We have previously shown that exposing young mice to a concept diet (Nuturis^®^; Nutricia Research) containing PL derived from the MFGM fraction, coated around large lipid droplets, prevented fat accumulation and improved metabolic profile in adulthood^(^ ^23^ ^,^ ^24^ ^)^. These data indicated that exposure to an altered dietary lipid structure in early life may be a key determinant of later life metabolic health. Thus far, however, it is not known how specific aspects of the Nuturis^®^ concept, that is, lipid droplet size and/or coating, may contribute to these later life beneficial effects. The objective of the present study was to determine whether large droplet size and/or coating are responsible for the previously found long-term protective effects against adult adiposity. To this end, we used a validated mouse model for nutritional programming^(^ ^25^ ^)^ in which young pre-weaning mice were exposed to intervention diets comprising different features of the Nuturis^®^ concept. After the early diet intervention, all mice were switched to a Western-style diet (WSD) and body composition development was monitored.

## Methods

### Animals and study design

**Fig. 1:**
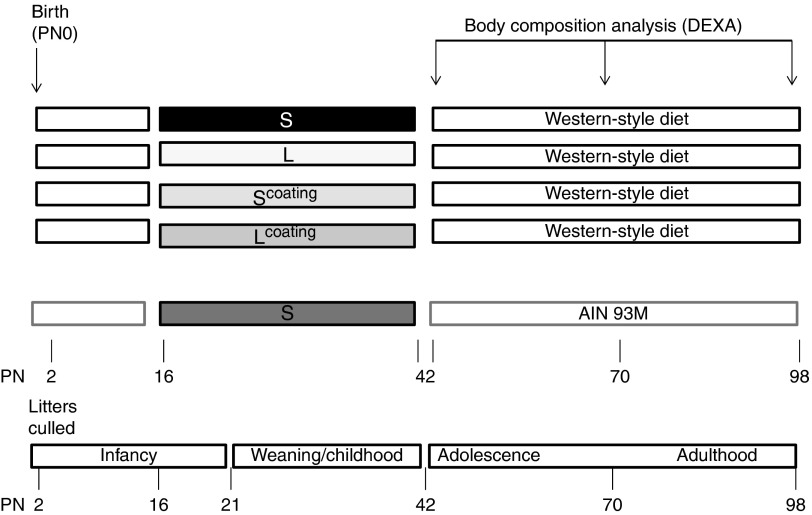
Study design from postnatal day (PN) 0–PN98. S, small droplets=Nutrilon; L, large droplets; S^coating^, small droplets coated by milk fat globule membrane (MFGM); L^coating^, large droplets coated by MFGM=Nuturis^®^; DEXA, dual-energy X-ray absorptiometry; IMF, infant milk formula; AIN93M, American Institute of Nutrition-93.

All experimental procedures were approved by the Animal Experimental Committee (DEC Consult) and complied with the principles of good laboratory animal care. The animals were housed at facilities of the Wageningen University and Research Centre in a 12 h light–12 h dark cycle (light on 06.00 hours=Zeitgeber time 0 h). Room temperature and humidity were controlled (21 (sem 2)°C and 50 (sem 5) %, respectively). During the entire protocol, food and water were available *ad libitum.* Between postnatal (PN) day 42 and 98, food intake was measured per cage and body weight (BW) was determined per litter before weaning and individually after weaning twice a week. Male and female C57BL/6J mice were time mated. Dams were assigned to the American Institute of Nutrition-93 (AIN93G) diet during pregnancy and lactation. After birth, litters were culled to four male and two female pups and randomly cross-randomised. Litters were randomly allocated to one of the experimental diets (see below) from PN16 until PN42. At weaning on PN21, male pups were housed in pairs and continued on their respective diets until PN42. From PN42 until dissection at PN98, animals were exposed to a WSD (20 %, w/w fat) (Fig. 1). To control for the effects of the WSD challenge in adolescence and adulthood, a reference (REF) group was included, which was fed the control IMF diet from PN15 to PN42 and the AIN93M diet from PN42 to PN98.

### Experimental diets

All experimental diets were semi-synthetic (Research Diet Services; Table 1) and had macronutrient and micronutrient compositions according to the AIN formulation of AIN93G-purified diets for laboratory rodents. The experimental diets contained 28·3 %, w/w IMF powder complemented with protein and carbohydrates to meet rodent nutrient requirements. The fat fraction was completely derived from the IMF. The detailed composition and processing procedure of the diets have been described previously^(^ ^24^ ^)^. The diets were provided as a dough in order to preserve the lipid structure of the products.

**Table 1:** Protein, lactose, lipid and phospholipid (PL) content (g), analysed per 100 g powder. Average of lipid droplet size (μm) of the different diets based on repeated productions

	Protein	Lactose	Lipids	PL	Mode*	% 2–12 μm†
S	10·4	50·7	25·4	0·05	0·4	4·8
L	9·5	50·2	25·4	0·05	4·4	52·7
S^coating^	10·1	47·3	24·9	0·4	0·4	2·9
L^coating^	9·7	48·3	25·4	0·5	3·2	56·0

S, small droplets; L, large droplets; S^coating^, small droplets coated by milk fat globule membrane (MFGM); L^coating^, large droplets coated by MFGM.

* Peak diameter of particle size distribution.

† Sum volume of particles of 2–12 mm (% total volume of all particles).

The experimental diets differed according to lipid droplet size (small (S) *v*. large (L)) and presence or absence of MFGM as lipid droplet coating: S (control IMF diet), L, S^coating^ and L^coating^ (Nuturis^®^ IMF diet). The MFGM originated from butter serum (SM 2; Corman Food Industry). In the production of anhydrous milk fat from cream, the aqueous streams are a by-product, rich in milk PL, present as MFGM fragments and referred to as butter serum^(^ ^26^ ^)^.

The oil blend of IMF was a mixture of oils such as rapeseed, sunflower, fish, coconut and palm oil. After IMF production, protein, lactose, lipid, PL concentrations and droplet size were measured (Table 1). The protein content was analysed according to the Dumas principle; the used *N*-factor was 6·25 (ISO14891 IDF 185, 2002). The lactose content was determined by an enzymatic method based on Nederlands Normalisatie-Instituut (NEN) 6853, 1989. Lipid content was determined according to the Van Gulik method (ISO11870, 2000). PL content was analysed with P-NMR by Spectral Services. The particle size distribution was determined by laser light scattering. The measurements were performed at 20°C, using a refractive index for fat of 1·46 and 0·01 absorption. The refractive index for the dispersant (water) of 1·33 was used (Mastersizer 2000, Hydro 2000G; Malvern Instruments Limited).

The WSD consisted of 20 %, w/w fat (3 %, w/w soya oil; 17 %, w/w lard and 0·1 %, w/w cholesterol).

### Body composition

Body composition was repeatedly measured between PN42 and 98 by dual-energy X-ray absorptiometry scan under general anaesthesia (isoflurane–N_2_O–O_2_) using a PIXImus imager (GE Lunar). Relative fat mass (FM) was calculated as the percentage FM (%FM) of total BW. Between PN42 and PN98, BW, lean body mass (LBM), FM and %FM gain (*δ*BW, *δ*LBM, *δ*FM and *δ*%FM, respectively) were calculated by subtracting PN42 values from PN98.

### Blood sampling and dissection

On PN98, mice were anaesthetised (isoflurane–N_2_O–O_2_) after fasting overnight and killed by bleeding (eye extraction) and cervical dislocation. Blood samples were collected in K3EDTA-coated 1-ml micro tubes. Plasma was obtained by centrifugation at 1350 ***g*** for 12 min at 4°C and stored at −80°C. The liver, pancreas, *musculus tibialis* and brain as well as epididymal (EPI), retroperitoneal (RP), perirenal (peri) and inguinal (ING) white adipose tissue (WAT) depots were dissected, weighed, snap-frozen in liquid N_2_ and stored at −80°C.

### Plasma analyses

Plasma glucose concentration was measured colorimetrically (glucose oxidase-phenol and 4 aminophenazone (GOD-PAP) method; Roche Diagnostics) and analysed using a micro plate imaging system (Bio-Rad Laboratories, Inc.). Insulin concentration was measured using ELISA (DRG) according to the manufacturer’s protocol. Homoeostasis model assessment of insulin resistance as calculated from fasting plasma glucose and insulin concentrations (glucose (mmol/l)×insulin (pmol/l)/22·5) as an indirect measure of insulin sensitivity. Total cholesterol, HDL-cholesterol, LDL-cholesterol, VLDL-cholesterol and TAG concentrations were determined colorimetrically after enzymatic conversion using a Roche Hitachi 717 analyzer (Reinier de Graaf Laboratory).

### Gene expression in retroperitoneal white adipose tissue

Total RNA was isolated from the RP WAT using Trizol–chloroform (Invitrogen) and purified using a RNeasy mini kit (Qiagen Benelux B.V.). Contaminating genomic DNA was removed with the RNase-free DNase set (Qiagen Benelux B.V.). Quality and quantity of RNA were determined by NanoDrop 2000 (Thermo Scientific) and Bioanalyzer (Agilent). Complementary DNA (cDNA) was synthesised using the iScript cDNA synthesis kit (Bio-Rad) according to the manufacturer’s instructions; 9·4 ng of RP fat depot was used as input for each quantitative PCR (qPCR) reaction. 5× Hot FIREPol Evagreen qPCR mix Plus (Bio-Connect) was used according to the manufacturer’s instructions and qPCR was accomplished with a 7900HT Fast Real-Time PCR System (Applied Biosystems). *Leptin* (marker adiposity), mesoderm-specific transcript/paternal expressed gene 1 (*mest/peg1*; marker adipocyte size) and preadipocyte factor 1 (*Pref1*; marker preadipocytes) mRNA expressions were measured. Normalisation of qPCR data was achieved using qBase PLUS (Biogazelle). qBase is a free programme for the analysis of qPCR data, and for this experiment several housekeeping genes (*calnexin, ribosomal protein gene L19, ribosomal protein S29*) were used to normalise the qPCR data. For detailed description, see the published article of Hellemans *et al*.^(^ ^27^ ^)^. The primer sequences are depicted in Table 2.

**Table 2:** Primer sequences

Gene name	NCBI reference number	Forward primer	Reverse primer
*Leptin*	NM_008493.3	AGGATGACACCAAAACCCTCAT	AGTCCAAGCCAGTGACCCTCT
*Mest/peg1*	NM_008590.1	TCAGTGACAAGCCGAGACCA	GTTGATTCTGCGGTTCTGGAG
*Pref1*	NM_010052.5	TGCGAGGCTGACAATGTCTG	ATGCACTGCCATGGTTCCTT
Reference genes			
*Canx*	NM_007597.3	AGAGCTCAGCCTGGATCAATTC	TTGTAGTCCTCTCCACACTTATCTGG
*Rpl19*	NM_009078.2	TTGCCTCTAGTGTCCTCCGC	CTTCCTGATCTGCTGACGGG
*Rps29*	NM_009093.2	AGTCACCCACGGAAGTTCGG	GTCCAACTTAATGAAGCCTATGTCCTT

*Mest/Peg1*, mesoderm-specific transcript/paternal expressed gene 1; *Pref1*, preadipocyte factor 1; *Canx*, calnexin; *Rpl19*, ribosomal protein gene L19; *Rps29*, ribosomal protein S29.

### Statistical analyses

Statistical analyses were performed using SPSS 19.0 (SPSS Benelux). Effects of diets on body composition, organ weights, plasma and gene expression parameters were analysed using mixed effects regression models, including droplet size and coating and size by coating interaction as fixed effects. In all regression models, the correlation among animals within the same cage was accounted for by their sharing a common random effect. Pairwise comparisons were adjusted for multiple comparisons, using the least square difference (LSD) approach. Data are shown as mean values with their standard errors. Differences were considered significant when *P*<0·05 and a trend was defined at *P*<0·1.

## Results

### Programming of adult food intake and body composition by dietary lipid structure

**Fig. 2:**
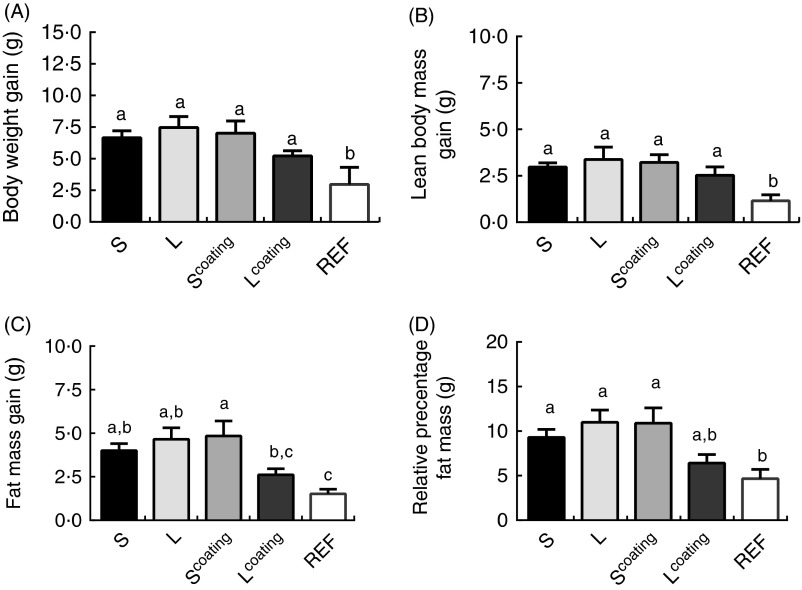
Relative body composition at postnatal day (PN) 98. (A) Body-weight gain. (B) Lean body mass. (C) Fat mass gain. (D) Relative percentage fat mass. Mice were fed different infant milk formula concepts, including small (S; *n* 12) *v*. large (L; *n* 12) lipid droplets, or with milk fat globule membrane as lipid droplet coating (coating) and challenged with a Western-style diet from PN42 till PN98. The reference (REF) group is the unchallenged (REF; *n* 12). Values are means, with their standard errors. ^a,b,c^ Mean values with unlike letters were significantly different (*P*<0·05). S=Nutrilon; L^coating^=Nuturis^®^.

Average energy intake from PN42 to PN98 was comparable between groups. To control for the effects of the WSD challenge, a REF group was included. At day 98, BW, LBM, FM and %FM of the REF group were lower than all other groups fed WSD (Fig. 2). However, FM of REF mice was significantly lower (approximately 64 %) compared with S, L and S^coating^ groups fed the WSD, but not significantly different from FM found in the L^coating^ group that showed comparable FM gain with the REF group.

At day 98, mice that received the concept diet containing MFGM coated around large lipid droplets (L^coating^, Nuturis^®^) showed significantly reduced FM compared with a diet containing small lipid droplets with comparable MFGM surface coating (*P*=0·05; Fig. 2; analysed using LSD) and a trend when compared with a diet containing large lipid droplets only (FM gain *P*=0·072 and %FM *P*=0·059, respectively).

BW was not significantly different between the four groups, although trends supported the above-summarised differences: BW (*F*=2161, *P*=0·157), LBM (*F*=1·074, *P*=0·312), FM (*F*=3·620, *P*=0·072) and %FM (*F*=3·646, *P*=0·071) at PN98 (analysed by a mixed effect regression model).

### Programming effects of dietary lipid structure in early-life diet on adult organ weights

The weight of the EPI fat depot was lower in mice fed L^coating^ in early life compared with small lipid droplets with or without MFGM surface coating (S, *P*=0·045 and S^coating^, *P*=0·05, respectively; Table 3) and a trend was observed when compared with large lipid droplets only (L; *P*=0·071). The ING fat depot weight was lower in L^coating^ mice (Nuturis^®^) compared with S^coating^ (*P*=0·018; analysed using LSD) and a trend was observed when compared with large lipid droplets only (L; *P*=0·066). The weight of the peri fat depot was decreased in L^coating^ mice compared with L (*P*=0·049) and a trend was detected when compared with S^coating^ (*P*=0·053). The weight of the liver was higher in S^coating^ and L compared with S (*P*=0·047 and *P*=0·021, respectively) and a trend was observed when compared with L^coating^ (*P*=0·073).

**Table 3:** Average weight of white adipose tissue (WAT) depots and organs of mice fed different infant milk formula diets and challenged with a Western-style diet from PN42 till PN98 (Mean values with their standard errors; *n* 12 per diet group)

	S		L		S^coating^		L^coating^	
	Mean	sem	Mean	sem	Mean	sem	Mean	sem
WAT								
Epididymal (mg)	998^a^	85	958^a,b^	107	989^a^	118	633^b^	77
Retroperitoneal (mg)	310	30	313	38	319	44	204	30
Inguinal (mg)	459^a,b^	31	498^a,b^	47	552^a^	66	338^b^	36
Perirenal (mg)	49^a,b^	7·2	60^a^	10	59^a,b^	9·6	28^b^	9·2
Liver (g)	1·4^a^	0·0	1·1^b^	0·1	1·2^b^	0·0	1·2^a,b^	0·0
Pancreas (mg)	153	6·1	170	12	164	7·1	147	6·5
*Musculus tibialis* (mg)	49	1·5	49	1·4	49	0·9	49	1·8
Brain (mg)	423	4·3	417	6·7	421	4·2	406	12

S, small droplets; L, large droplets; S^coating^, small droplets coated by milk fat globule membrane (MFGM); L^coating^, large droplets coated by MFGM. ^a,b^ Mean values with unlike superscript letters were significantly different (*P*<0·05).

### Effects of dietary lipid structure in early-life diet on adult plasma parameters

At day 98, plasma parameters were only moderately affected by the WSD itself, as indicated by the results of the REF group: only glucose concentrations were 44 % lower in REF mice fed a standard rodent diet compared with the groups fed WSD (*P*<0·05, data not shown). The multivariate analysis results are depicted in the supplements (online Supplementary Table S1; analysed using LSD).

### Programming of markers for adiposity, adipocyte size and cell differentiation

**Fig. 3:**
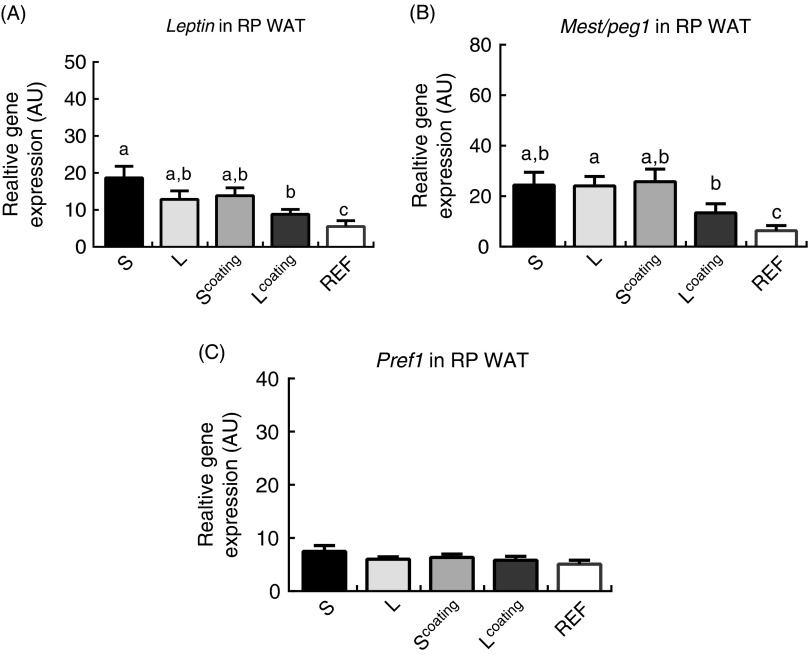
mRNA expressions in arbitrary units (AU) of *leptin* (A), mesoderm-specific transcript/paternal expressed gene 1 (*mest/peg1*) (B) and preadipocyte factor 1 (*pref1*) (C) in retroperitoneal (RP) white adipose tissue (WAT) of mice fed different infant milk formula concepts, including small (S) *v*. large (L) lipid droplets, or with milk fat globule membrane as lipid droplet coating (coating) and challenged with a Western-style diet from postnatal (PN) 42 till PN98. Sample size is 12, 11, 10, 12, respectively. The reference (REF) group is the unchallenged (REF; *n* 10). Values are means, with their standard errors. ^a,b,c^ Mean values with unlike letters were significantly different (*P*<0·05). S=Nutrilon; L^coating^=Nuturis^®^.

At day 98, *leptin* expression was significantly lower in the L^coating^ group compared with the S control group (*P*=0·023; Fig. 3(a); analysed using LSD). Besides, *mest/peg1* expression in L^coating^ (Nuturis^®^) was decreased compared with L following the WSD challenge (*P*=0·043; Fig. 3(b)), but only a trend was seen for lower *mest/peg1* expression in L^coating^ compared with S^coating^ and S (*P*=0·053 and *P*=0·051, respectively).

## Discussion

The present study aimed to determine whether providing a diet in early life containing large lipid droplets, MFGM as lipid droplet coating or their combination, was responsible for the long-term protective effect against adiposity in a mouse model. Our results clearly indicate that the combination of a large droplet size and MFGM present as a lipid droplet coating was essential for prevention of excessive body FM accumulation in adult life. Neither an increase in droplet size alone nor MGFM coating of small lipid droplets exerted any beneficial programming of adult body composition. These data suggest that the complex structure of large lipid droplet size consisting of a TAG core surrounded by a trimembrane layer as present in HM may significantly contribute to its reported protective effect against obesity in later life. These outcomes support a role of lipid matrix beyond the FA composition in dietary lipid quality in early life linked to later life health.

The mechanism(s) underlying the reduction in fat accumulation by early feeding with the Nuturis^®^ diet is not completely clear. As food intake between groups was comparable, differences in food intake cannot explain the observed adult phenotype. The structural aspects of the Nuturis^®^ concept, which bring its nutrient matrix closer to the HM structure, may affect digestion and absorption, and thereby modulate the postprandial response and FA metabolism^(^ ^16^ ^)^. We hypothesise that differences in postprandial lipid handling may programme metabolic homoeostasis and energy balance. This could impact the development and function of metabolic organs including intestine, pancreas, liver, brain and WAT, and thereby influence later life metabolic health. Previous studies have indeed shown that the lipid structure impacts gastric emptying, lipid absorption and digestion kinetics as well as the postprandial handling of lipids^(^ ^21^ ^,^ ^22^ ^)^. For example, in adult rats, HM-derived lipid droplets coated by MFGM alters the TAG appearance compared with small droplets with proteins at the interface^(^ ^28^ ^)^. It clearly shows the importance of lipid droplet size as well as the nature of the surface coating, both impacting physical digestion and absorption rate. In general, lipids will either be targeted for *β*-oxidation or stored in WAT depending on these differences in kinetics and metabolic fate. Clearly, in obese people, lipid handling has been favoured towards lipid storage instead rather than towards *β*-oxidation^(^ ^29^ ^)^. Absorption and digestion kinetics are different between adults and infants related to the digestive immaturity of the infant^(^ ^30^ ^,^ ^31^ ^)^. The latter provides the possibility for lipids to be a major source of energy as well as education for the developing metabolism, affecting growth and metabolic development. We speculate that in particular the combination of lipid droplet size and its surface composition of PL originating from MFGM fraction as present in Nuturis^®^ during infancy may impact metabolic development and programme later life (metabolic) health. As a consequence, the adult metabolic homoeostasis may be more flexible to handle various lifestyle challenges, for example, a diet higher in (SFA) fats. Fat oxidation could be one of the targets of programming, but others are also possible, including glucose homoeostasis, fat absorption kinetics and postprandial physiology.

Future research is required to assess whether differential digestion and absorption kinetics induced by lipid structure of Nuturis^®^ (L^coating^) indeed underlie the long-term effects found in the present study. Recent imaging experiments comparing Nuturis^®^ lipid droplets indeed confirm the notion that the lipid structure is closer to that observed in HM (Gallier *et al*., unpublished results).

One of the possible mechanisms that could contribute to the observed protective effect of Nuturis^®^ on body fat accumulation is reduced lipid storage capacity and a decline in lipogenesis in WAT. Previously, we found a reduced expression of *PPAR* *γ* (related to lipogenesis) in mice fed Nuturis^®^ compared with mice fed control diet (S) before the WSD challenge^(^ ^23^ ^)^. Indeed, WAT development during the PN period may determine the size and function of WAT in later life^(^ ^32^ ^)^. The total adipocyte number increases in early life and a constant fat cell number is only established by early adulthood^(^ ^33^ ^,^ ^34^ ^)^. FM changes in adulthood are mostly driven by changes in adipocyte cell volume and not cell number^(^ ^34^ ^)^. *Mest/peg1*, a marker for adipocyte size, is highly expressed in WAT and inhibits the Wnt signalling pathway, which stimulates the differentiation of stem cells in preadipocytes^(^ ^34^ ^–^ ^38^ ^)^. We found that early-life feeding with Nuturis^®^ could lower the expression of *mest/peg1* in RP WAT, although not significant compared with our control diet containing small lipid droplets with or without MFGM coating. This observation indeed supports the reduced FM seen in Nuturis^®^-fed mice. FM also strongly corresponded with *leptin* expression levels in RP WAT, particularly in Nuturis^®^-fed mice (L^coating^; *R* 0·732, *P*=0·000). Plasma leptin concentrations are positively correlated to FM in both humans and rodents and implicated in energy homoeostasis^(^ ^39^ ^–^ ^41^ ^)^. Detailed analysis of adipose tissue to gain further insights into adipose tissue function is one of the approaches needed to understand the underlying mechanisms for the observed protective effect of Nuturis^®^ feeding in early life. The expression of genes implicated in lipogenesis and lipolysis cannot be easily differentiated from the present study design, because the observed effect is a combination between programming and a WSD effect. Rather, a study with a specific experimental design is required to obtain mechanistic information on lipogenesis and lipolysis. Studies to obtain more detailed information on lipogenesis and lipid storage in WAT as well as *in vivo* energy expenditure are needed to determine whether these possibilities are indeed the underlying mechanisms.

MFGM supplementation to IMF has been proven to be safe and well tolerated^(^ ^42^ ^)^. Recent studies by Timby *et al*.^(^ ^43^ ^)^ suggest beneficial effects of adding MFGM lipid products to IMF in infants on specific immune and cognitive outcomes. Cognitive development and serum cholesterol concentrations in MFGM-enriched formula-fed infants were closer to breast-fed infants at 12 months of age^(^ ^19^ ^,^ ^44^ ^)^. Although it was hypothesised that these effects could have sustained effects on adult cholesterol metabolism, as well as on the risk for CVD^(^ ^20^ ^)^. No effects were reported on growth or long-term health outcomes. In a recently published editorial, Greer & Kleinman^(^ ^45^ ^)^ raised the question whether adding MFGM combined with lower protein and energy contents to IMF could bring IMF closer to HM composition and health benefits attributed to breast-feeding. Our results clearly show that adding MFGM as a coating to current IMF containing small lipid droplets, only the combination of a large lipid droplet size and the MFGM coating showed beneficial effects on later life FM gain. Notably, in the studies by Timby *et al.* ^(^ ^19^ ^,^ ^20^ ^,^ ^43^ ^)^ MFGM was added to an IMF that was lower in both energy as well as protein compared with the IMF used to construct the rodent diets. Therefore, the observed effects found in Timby *et al.* could also, at least in part, be explained by the combination of these nutrient composition and energy differences instead of the MFGM supplementation alone. Clinical studies testing the effect of Nuturis^®^ on growth and body composition development are currently on-going (clinical trial registry NCT01609634 and the Dutch Trial Register NTR3683).

Although we found reduced adiposity in adult life after feeding a diet with an altered lipid structure in early life, we did not observe significant improvements in metabolic parameters as far as tested. This could be explained, at least in part, by the fact that the WSD challenge was rather mild as it did not generate major disturbances in plasma lipid or glucose homoeostasis compared with the unchallenged REF group, nor resulted in hyperphagia or overt obesity. The choice of this relatively mild obesogenic diet was based on the assumption that this would mimick a modern Western lifestyle more closely. Indeed, the WSD challenge in our model did not induce hyperphagia as is commonly seen with more extreme WSD diets in the literature^(^ ^46^ ^,^ ^47^ ^)^. To assess whether the Nuturis^®^ diet may protect against the development of metabolic risk factors, it would be interesting to extend the duration of the mild WSD challenge and/or use a more severe dietary challenge in future studies.

In conclusion, a diet containing the combination of large lipid droplets and a coating with MFGM mimicking the lipid droplet architecture as present in mammalian milk prevents body fat accumulation when challenged with a moderate WSD during adolescence and adulthood. These observations confirm the notion that the complex lipid structure beyond the composition of lipids may be an important factor in the early diet that determines growth and development of body composition and impacts long-term (metabolic) health. Future experimental and clinical studies are required to confirm the protective effect of this concept in humans and to gain more detailed insights about the underlying mechanisms.

## Supplementary material

For supplementary material accompanying this paper visit http://dx.doi.org/10.1017S0007114516001082. click here to view supplementary material

## References

[1] Dewey KG (2003) Is breastfeeding protective against child obesity? J Hum Lact 19, 9–18. 12587638 10.1177/0890334402239730

[2] Harder T, Bergmann R, Kallischnigg G, et al (2005) Duration of breastfeeding and risk of overweight: a meta-analysis. Am J Epidemiol 162, 397–403. 16076830 10.1093/aje/kwi222

[3] Owen CG, Martin RM, Whincup PH, et al (2006) Does breastfeeding influence risk of type 2 diabetes in later life? A quantitative analysis of published evidence. Am J Clin Nutr 84, 1043–1054. 17093156 10.1093/ajcn/84.5.1043

[4] Ryan AS (2007) Breastfeeding and the risk of childhood obesity. Coll Antropol 31, 19–28. 17598382

[5] Arenz S, Ruckerl R, Koletzko B, et al (2004) Breast-feeding and childhood obesity – a systematic review. Int J Obes Relat Metab Disord 28, 1247–1256. 15314625 10.1038/sj.ijo.0802758

[6] Burrin D, Stoll B & Moore D (2013) Digestive physiology of the pig symposium: intestinal bile acid sensing is linked to key endocrine and metabolic signaling pathways. J Anim Sci 91, 1991–2000. 23729782 10.2527/jas.2013-6331 PMC3984497

[7] Lönnerdal B (2003) Nutritional and physiologic significance of human milk proteins. Am J Clin Nutr 77, 1537S–1543S. 12812151 10.1093/ajcn/77.6.1537S

[8] Innis SM (2007) Human milk: maternal dietary lipids and infant development. Proc Nutr Soc 66, 397–404. 17637092 10.1017/S0029665107005666

[9] Carlson SE (2009) Early determinants of development: a lipid perspective. Am J Clin Nutr 89, 1523S–1529S. 19321568 10.3945/ajcn.2009.27113G PMC2677004

[10] Gallier S, Gragson D, Cabral C, et al (2010) Composition and fatty acid distribution of bovine milk phospholipids from processed milk products. J Agric Food Chem 58, 10503–10511. 20828196 10.1021/jf101878d PMC4243513

[11] Fong BY, Norris CS & MacGibbon AK (2007) Protein and lipid composition of bovine milk-fat-globule membrane. Int Dairy J 17, 275–288.

[12] Rueda R (2014) The role of complex lipids in attaining metabolic health. Curr Cardiovasc Risk Rep 8, 1–8.

[13] Michalski MC (2009) Specific molecular and colloidal structures of milk fat affecting lipolysis, absorption and postprandial lipemia. Eur J Lipid Sci Technol 111, 413–431.

[14] Michalski M, Briard V, Michel F, et al (2005) Size distribution of fat globules in human colostrum, breast milk, and infant formula. J Dairy Sci 88, 1927–1940. 15905422 10.3168/jds.S0022-0302(05)72868-X

[15] Michalski MC, Calzada C, Makino A, et al (2008) Oxidation products of polyunsaturated fatty acids in infant formulas compared to human milk – a preliminary study. Mol Nutr Food Res 52, 1478–1485. 18792926 10.1002/mnfr.200700451

[16] Fave G, Coste T & Armand M (2004) Physicochemical properties of lipids: new strategies to manage fatty acid bioavailability. Cell Mol Biol (Noisy-le-Grand) 50, 815–831. 15672466

[17] Spitsberg VL (2005) Invited review: bovine milk fat globule membrane as a potential nutraceutical. J Dairy Sci 88, 2289–2294. 15956291 10.3168/jds.S0022-0302(05)72906-4

[18] Vickers MH, Guan J, Gustavsson M, et al (2009) Supplementation with a mixture of complex lipids derived from milk to growing rats results in improvements in parameters related to growth and cognition. Nutr Res 29, 426–435. 19628110 10.1016/j.nutres.2009.06.001

[19] Timby N, Domellöf E, Hernell O, et al (2014) Neurodevelopment, nutrition, and growth until 12 mo of age in infants fed a low-energy, low-protein formula supplemented with bovine milk fat globule membranes: a randomized controlled trial. Am J Clin Nutr 99, 860–868. 24500150 10.3945/ajcn.113.064295

[20] Timby N, Lönnerdal B, Hernell O, et al (2014) Cardiovascular risk markers until 12 mo of age in infants fed a formula supplemented with bovine milk fat globule membranes. Pediatr Res 76, 394–400. 25116230 10.1038/pr.2014.110

[21] Armand M, Hamosh M, Mehta NR, et al (1996) Effect of human milk or formula on gastric function and fat digestion in the premature infant 1. Pediatr Res 40, 429. 8865280 10.1203/00006450-199609000-00011

[22] Armand M, Pasquier Brr, Andre M, et al (1999) Digestion and absorption of 2 fat emulsions with different droplet sizes in the human digestive tract. Am J Clin Nutr 70, 1096–1106. 10584056 10.1093/ajcn/70.6.1096

[23] Oosting A, Kegler D, Wopereis HJ, et al (2012) Size and phospholipid coating of lipid droplets in the diet of young mice modify body fat accumulation in adulthood. Pediatr Res 72, 362–369. 22850409 10.1038/pr.2012.101

[24] Oosting A, van Vlies N, Kegler D, et al (2013) Effect of dietary lipid structure in early postnatal life on mouse adipose tissue development and function in adulthood. Br J Nutr 111, 215–226. 23845308 10.1017/S0007114513002201

[25] Oosting A, Kegler D, Boehm G, et al (2010) *n-*3 Long-chain polyunsaturated fatty acids prevent excessive fat deposition in adulthood in a mouse model of postnatal nutritional programming. Pediatr Res 68, 494–499. 20724957 10.1203/PDR.0b013e3181f74940

[26] Vanderghem C, Bodson P, Danthine S, et al (2010) Milk fat globule membrane and buttermilks: from composition to valorization. Biotechnol Agron Soc Environ 14, 485.

[27] Hellemans J, Mortier G, De Paepe A, et al (2007) qBase relative quantification framework and software for management and automated analysis of real-time quantitative PCR data. Genome Biol 8, R19. 17291332 10.1186/gb-2007-8-2-r19 PMC1852402

[28] Michalski M-C, Soares AF, Lopez C, et al (2006) The supramolecular structure of milk fat influences plasma triacylglycerols and fatty acid profile in the rat. Eur J Nutr 45, 215–224. 16432662 10.1007/s00394-006-0588-9

[29] Bourlieu C & Michalski M-C (2015) Structure-function relationship of the milk fat globule. *Curr Opin Clin Nutr Metab Care* 18, 118–127. 10.1097/MCO.0000000000000138 25581036

[30] Abrahamse E, Minekus M, van Aken GA, et al (2012) Development of the digestive system – experimental challenges and approaches of infant lipid digestion. Food Dig 3, 63–77. 23293684 10.1007/s13228-012-0025-x PMC3528963

[31] Michalski MC, Genot C, Gayet C, et al (2013) Multiscale structures of lipids in foods as parameters affecting fatty acid bioavailability and lipid metabolism. Prog Lipid Res 52, 354–373. 23624223 10.1016/j.plipres.2013.04.004

[32] Budge H, Sebert S, Sharkey D, et al (2009) Session on ‘Obesity’. Adipose tissue development, nutrition in early life and its impact on later obesity. Proc Nutr Soc 68, 321–326. 19490741 10.1017/S0029665109001402

[33] Knittle J, Timmers K, Ginsberg-Fellner F, et al (1979) The growth of adipose tissue in children and adolescents. Cross-sectional and longitudinal studies of adipose cell number and size. J Clin Invest 63, 239–246. 429551 10.1172/JCI109295 PMC371945

[34] Spalding KL, Arner E, Westermark PO, et al (2008) Dynamics of fat cell turnover in humans. Nature 453, 783–787. 18454136 10.1038/nature06902

[35] Takahashi M, Kamei Y & Ezaki O (2005) Mest/Peg1 imprinted gene enlarges adipocytes and is a marker of adipocyte size. Am J Physiol Endocrinol Metab 288, E117–E124. 15353408 10.1152/ajpendo.00244.2004

[36] Hwajin J, Suk KL & Eek-hoon J (2011) Mest/Peg1 inhibits Wnt signalling through regulation of LRP6 glycosylation. Biochem J 436, 263–269. 21375506 10.1042/BJ20101512

[37] Nikonova L, Koza RA, Mendoza T, et al (2008) Mesoderm-specific transcript is associated with fat mass expansion in response to a positive energy balance. FASEB J 22, 3925–3937. 18644838 10.1096/fj.08-108266 PMC2574032

[38] Laudes M (2011) Role of WNT signalling in the determination of human mesenchymal stem cells into preadipocytes. J Mol Endocrinol 46, R65–R72. 21247979 10.1530/JME-10-0169

[39] Maffei M, Halaas J, Ravussin E, et al (1995) Leptin levels in human and rodent: measurement of plasma leptin and ob RNA in obese and weight-reduced subjects. Nat Med 1, 1155–1161. 7584987 10.1038/nm1195-1155

[40] Leibel RL (2002) The role of leptin in the control of body weight. Nutr Rev 60, S15–S19. 12403079 10.1301/002966402320634788

[41] Guo K-Y, Halo P, Leibel RL, et al (2004) Effects of obesity on the relationship of leptin mRNA expression and adipocyte size in anatomically distinct fat depots in mice. Am J Physiol Regul Integr Comp Physiol 287, R112–R119. 15001430 10.1152/ajpregu.00028.2004

[42] Billeaud C, Puccio G, Saliba E, et al (2014) Safety and tolerance evaluation of milk fat globule membrane-enriched infant formulas: a randomized controlled multicenter non-inferiority trial in healthy term infants. Clin Med Insights Pediatr 8, 51–60. 25452707 10.4137/CMPed.S16962 PMC4219856

[43] Timby N, Hernell O, Vaarala O, et al (2015) Infections in infants fed formula supplemented with bovine milk fat globule membranes. A randomized controlled trial. J Pediatr Gastroenterol Nutr 60, 384–389. 25714582 10.1097/MPG.0000000000000624

[44] Timby N, Lönnerdal B, Hernell O, et al (2014) Cardiovascular risk markers until 12 months of age in infants fed a formula supplemented with bovine milk fat globule membranes. *Pediatr Res* 76, 394–400. 10.1038/pr.2014.110 25116230

[45] Greer FR & Kleinman RE (2014) An infant formula with decreased weight gain and higher IQ: are we there yet? Am J Clin Nutr 99, 757–758. 24598153 10.3945/ajcn.114.084798

[46] Wei X, Sun B, Chen K, et al (2015) Ghrelin signaling in the ventral tegmental area mediates both reward-based feeding and fasting-induced hyperphagia on high-fat diet. Neuroscience 300, 53–62. 25967263 10.1016/j.neuroscience.2015.05.001

[47] Duval C, Thissen U, Keshtkar S, et al (2010) Adipose tissue dysfunction signals progression of hepatic steatosis towards nonalcoholic steatohepatitis in C57Bl/6 mice. Diabetes 59, 3181–3191. 20858684 10.2337/db10-0224 PMC2992781

