# Supplementary material for: Milk fat globule membrane coating of large lipid droplets in the diet of young mice prevents body fat accumulation in adulthood
Source: Br J Nutr. 2016 Apr 4;115(11):1930–7. doi: 10.1017/S0007114516001082 (PMC4863696; doi:10.1017/S0007114516001082)
Supplement: Supplementary file 1 [file S0007114516001082sup001.docx]

ONLINE SUPPLEMENTAL MATERIAL

Supplemental Table 1. Average fasting plasma parameters of mice fed different IMF diets and challenged with a WSD from PN42 till PN98

|  | |  | **S** | **L** | **S ^coating^** | **L ^coating^** |
| --- | --- | --- | --- | --- | --- | --- |
| **PN98** |  | |  |  |  |  |
| **Lipids** | **Total cholesterol (mmol/L** | | 3.6 ± 0.2 | 3.1 ± 0.3 | 3.2 ± 0.3 | 3.1 ± 0.2 |
|  | **Triglycerides (mmol/L** | | 0.8 ± 0.1^a^ | 0.7 ± 0.0^ab^ | 0.6 ± 0.1^b^ | 0.8 ± 0.1^ab^ |
|  | **HDL (mmol/L)** | | 1.9 ± 0.1 | 1.6 ± 0.2 | 1.6 ± 0.2 | 1.6 ± 0.1 |
|  | **LDL (mmol/L** | | 1.3 ± 0.1 | 1.2 ± 0.1 | 1.3 ± 0.2 | 1.2 ± 0.1 |
|  | **VLDL (mmol/L)** | | 0.4 ± 0.0^a^ | 0.3 ± 0.0^ab^ | 0.3 ± 0.0^b^ | 0.3 ± 0.0^ab^ |
| **Glucose homeostasis** | **Insulin (pmol/L)** | | 104 ± 21 | 96 ± 20 | 149 ± 39 | 88 ± 15 |
|  | **Glucose (mmol/L)** | | 15 ± 0.8 | 15 ± 1.9 | 17 ± 1.1 | 15 ± 1.2 |
|  | **HOMA-IR (mmol/L x pmol/L/2.25)** | | 76 ± 22 | 71 ± 14 | 117 ± 32 | 54 ± 10 |

Glucose PN98 n=10,9,11,10, respectively; Insulin PN98 n=8,9,7,9 respectively: HOMA-IR PN98 n=7,7,7,7 respectively; CHOL, TG, HDL, LDL and VLDL n=12 respectively. p < 0.05 is indicated by a different letter.
